# Death of Dr. F. W. Abbott

**Published:** 1901-05

**Authors:** 


### DEATH OF DR. F. W. ABBOTT.

Dr. Frank Wayland Abbott, U. of B., '66, died at his home, 523 Franklin Street, Buffalo, Tuesday, April 9, 1901, after a protracted illness, aged 59 years. Dr. Abbott for years has occupied a prominent place in the profession of medicine, distinguished for his skill, judgment, accuracy and integrity. He was one of the most prominent ophthalmologists and otologists in Buffalo, serving in that capacity on the staff of the General Hospital, on the Erie County Charity Eye, Ear and Throat Hospital, and as a member of the Board of Pension Surgeons. For many years he was chairman of the executive committee of the Buffalo General Hospital Training School for Nurses.

Dr. Abbott was a lay reader of the Episcopal Church and has done much excellent work in that capacity. He is survived by his widow and one son, Wayne Abbott. His funeral was held from St. Paul's Church, April 12, at 4 o'clock, Rev. J. A. Regester and Rev. G. B. Richards officiated. The bearers were Mr. Henry R. Howland, Dr. H. R. Hopkins, Dr. W. C. Phelps, Dr. Lorenzo Burrows, Dr. A. A. Hubbell, Dr. M. D. Mann, Dr. E. C. W. O'Brien and Dr. John Parmenter. The interment was in the family lot in Forest Lawn. Elsewhere in the Journal we publish the action of several bodies of which Dr. Abbott was a member.

